# Ultrasound-guided percutaneous retrieval of a dropped gallstone following laparoscopic cholecystectomy

**DOI:** 10.1259/bjrcr.20180002

**Published:** 2018-04-11

**Authors:** Benedict Thomson, Bhavin Kawa, Amanda Rabone, Yasser Abdul-Aal, Fazal Hasan, Paul Ignotus, Aidan Shaw

**Affiliations:** 1 Department of Interventional Radiology, Maidstone and Tunbridge Wells NHS Trust, Tunbridge Wells Hospital, Pembury, UK; 2 Department of General Surgery, Maidstone and Tunbridge Wells NHS Trust, Tunbridge Wells Hospital, Pembury, UK

## Abstract

Removal of intraabdominal dropped gallstones remains a challenging problem for both surgeon and radiologist. We describe in this report a novel, minimally invasive technique to successfully remove a dropped gallstone, causing recurrent intra-abdominal infection, from a patient who had undergone laparoscopic cholecystectomy.

## Summary

Dropped gallstones are a relatively common complication of laparoscopic cholecystectomy and most stones are retrieved intraoperatively.^1, 2^ In aminority of cases retained stones can induce a local inflammatory response leading to abscess formation within the abdomen.^3^ Surgical options available for the extraction of dropped gallstones carry increased risks of post-operative morbidity.^4^ We describe a novel, minimally invasive, ultrasound-guided technique to successfully remove a dropped gallstone, causing intraabdominal sepsis, from a patient who had undergone laparoscopic cholecystectomy.

## Clinical presentation

An 84-year-old Caucasian male underwent an elective laparoscopic cholecystectomy for symptomatic gallstones. Initially, the patient made a good post-operative recovery. However, over the next few months, he had repeated episodes of low-grade sepsis as well as loss of appetite and weight loss. He was readmitted to hospital 5 months post-procedure after developing fever, rigors and ongoing abdominal pain. On examination, he was tender on palpation of his right upper quadrant and initial blood tests showed elevated inflammatory markers.

## Imaging findings

**Figure 1.**
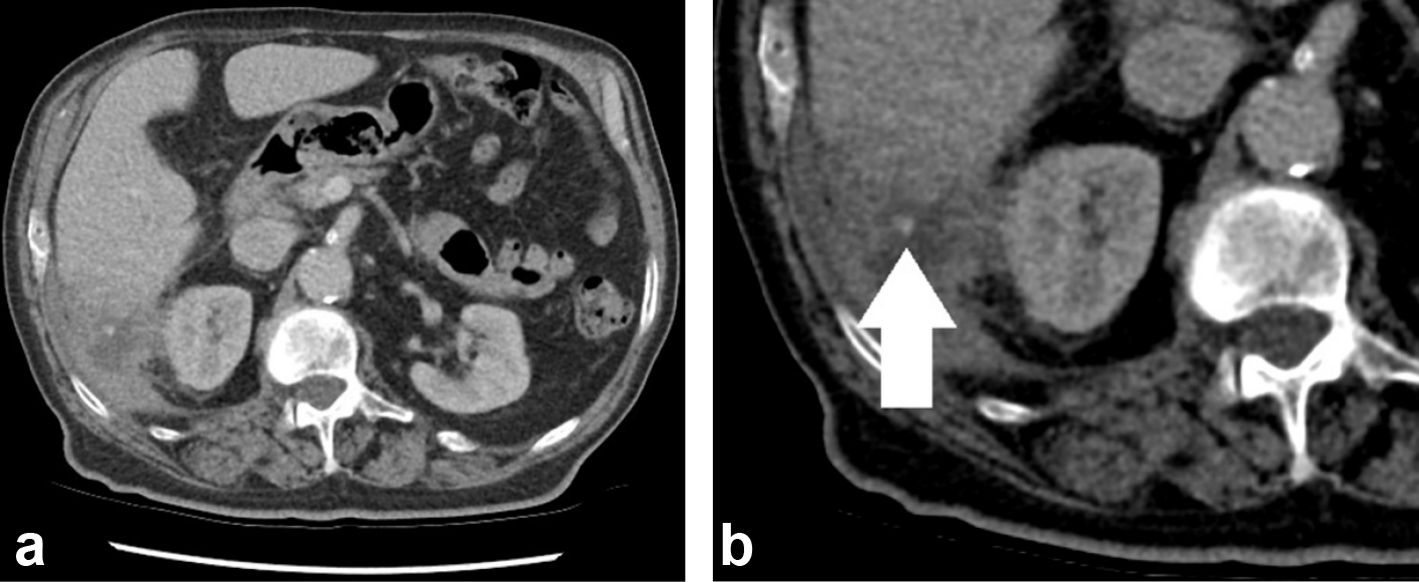
CT following intravenous contrast demonstrating the (a) perihepatic collection and (b) magnified view showing the 6 mm radiopaque gallstone (white arrow).

A CT scan of his abdomen and pelvis was performed which showed a complex 9 × 3.7 cm perihepatic collection within which a 6 mm high attenuating focus was identified consistent with a dropped gallstone (Figure 1).

**Figure 2.**
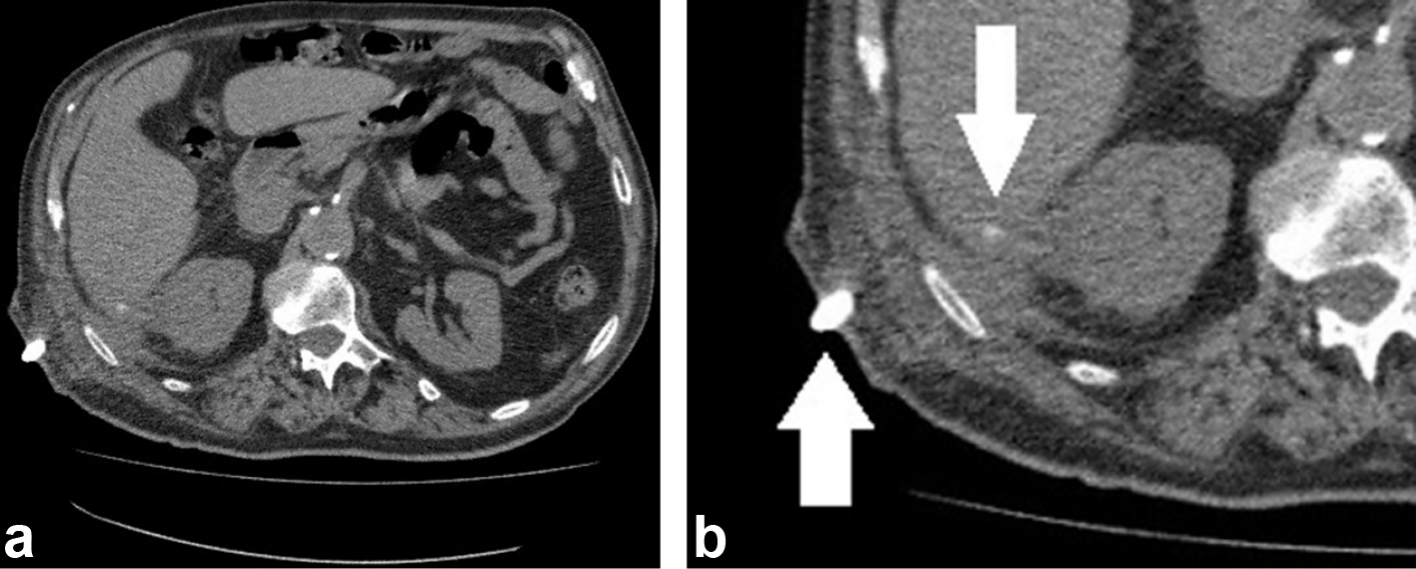
Unenhanced abdominal CT showing (a) partially resolved collection and (b) magnified view with the drain (↑) and the gallstone still visible (↓).

The patient was initially resuscitated with antibiotics and fluids, and then underwent successful ultrasound-guided percutaneous drainage of the collection with a 14 Fr pigtail catheter using Seldinger technique. Interval CT showed that the abscess had resolved. However, the dropped gallstone remained centred within the residual collection (Figure 2).

## Treatment

There was concern that a surgical approach to remove the gallstone would prove difficult and may result in severe bleeding from the liver capsule on tissue dissection. Given the repeated episodes of sepsis and weight loss, and after discussion with the patient and surgical colleagues, a more minimally invasive procedure involving percutaneous drainage and retrieval of the dropped stone was planned.

1 week following siting the pigtail drain and resolution of the sepsis, the patient was brought to the interventional suite. The procedure was performed under local anaesthetic and conscious sedation. Unfortunately, the night before the procedure, the pigtail drain fell out of the collection. However, the gallstone could be well visualised under ultrasound surrounded by soft tissue from the resolved collection. Fluoroscopy was also performed to see if this could assist with the procedure but the stone could not be identified.

**Figure 3.**
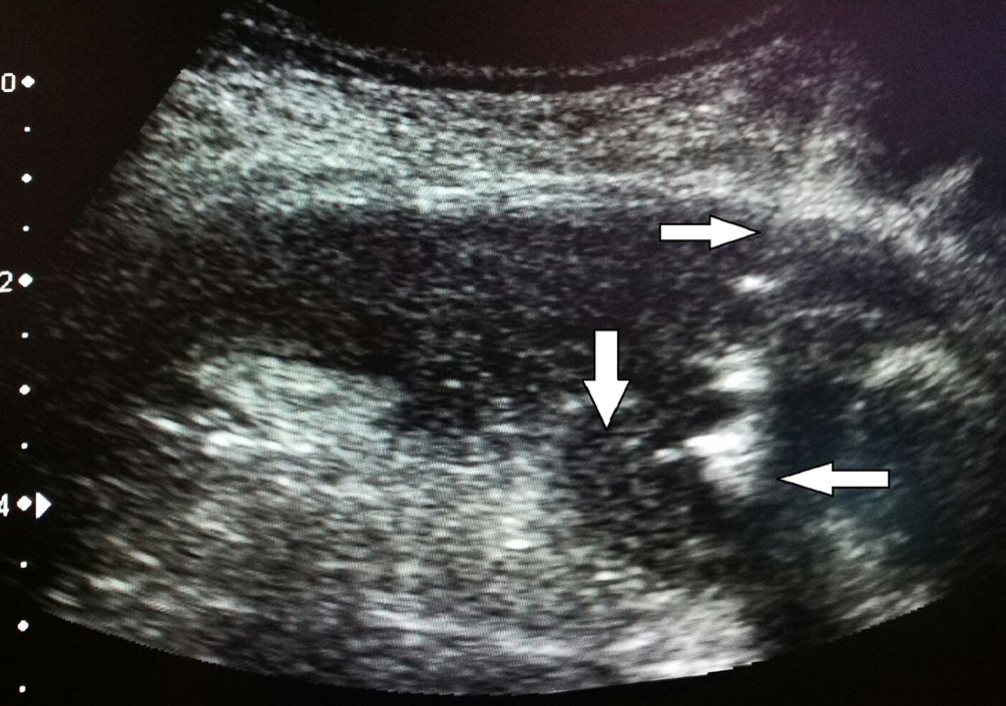
Ultrasound image shows the 18 G DTN needle (→) puncturing onto the hyperechoic gallstone (←) within the hypoechoic surrounding collection (↓).

An ultrasound-guided retrieval was, therefore, planned. Using ultrasound guidance, an 18G DTN needle (Cook Medical, Bloomington, IN) was placed onto the gallstone within the perihepatic region (Figure 3) through the same tract as the previous drain. A small volume of normal saline was injected to create a fluid space/iatrogenic collection around the gallstone. A short stiff guidewire was then passed and serial tract dilation was performed followed by insertion of a 5.5 cm 11 Fr vascular sheath.

**Figure 4.**
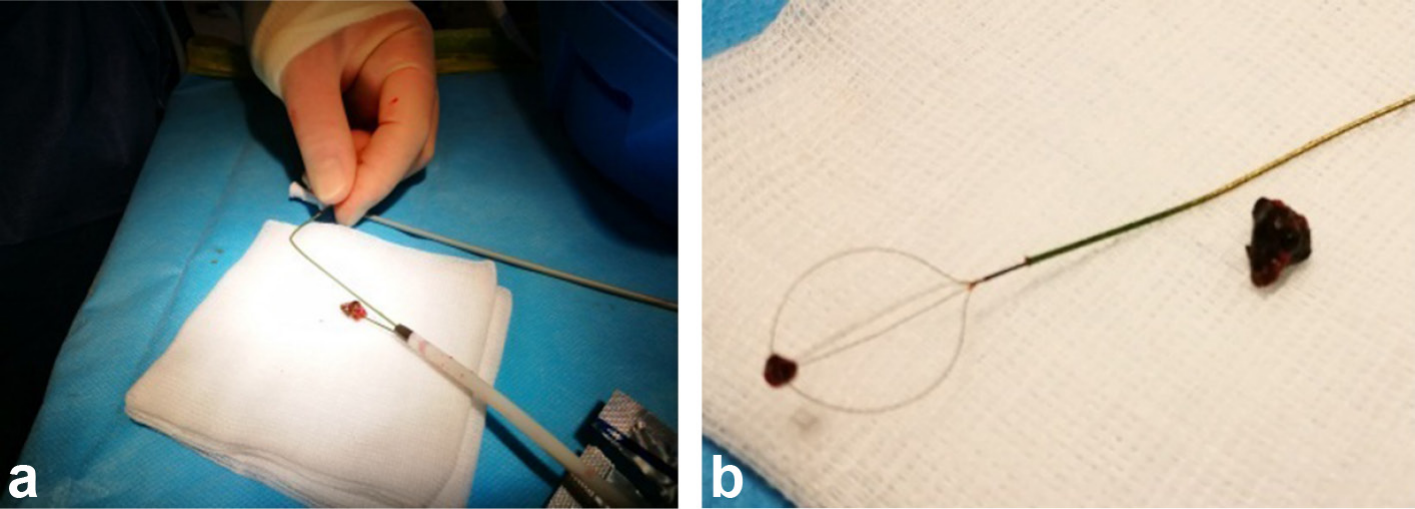
Gallstone successfully snared (a) and removed with the basket snare (b).

A 1.9 Fr Zero Tip^TM^ nitinol stone retrieval basket (Boston Scientific, Marlborough, MA) was then placed through the sheath. Although air had entered the fluid space, the 6 mm gallstone could still be visualised and was snared under ultrasound guidance. The gallstone was locked against the tip of the vascular sheath with the snare, and everything removed as one, successfully retrieving the gallstone whole with no residual calculus identified (Figure 4).

## Outcome and follow up

There were no periprocedural complications. The procedure was well tolerated and the patient was discharged from hospital a few days later. At follow up, the patient had improved clinically and biochemically with significant improvements in his appetite, weight, general well-being and inflammatory markers. As such no follow up ultrasound or imaging was performed.

## Discussion

Dropped gallstones are a relatively common complication of laparoscopic cholecystectomy, occurring in approximately 7% of procedures.^1, 2^ This is usually as a result of gallbladder perforation during dissection. Most are retrieved intraoperatively but it is estimated 2.4% are left in the peritoneum. The majority of retained stones are asymptomatic and only 0.08–0.3% of patients become symptomatic.^5, 6^

A dropped gallstone induces local inflammation initiating a low-grade granulomatous response. Pigment stones are thought to initiate the largest inflammatory response. In the majority of cases, the dropped stone forms a benign granulomatous deposit, which may be incidentally identified on ensuing cross-sectional imaging. However, in a minority of cases the inflammatory response will persist. This can lead to either an abscess, sinus tract or fistula. The patient will then become symptomatic. The stone may also erode through the peritoneal cavity and migrate to other areas of the body.^3^ Abscess formation is one of the most serious complications of dropped stones. The condition typically presents, as in our case, many months after the procedure.^7^

There are several surgical options for the extraction of dropped gallstones but these carry significantly increased risks of post-operative morbidity.^4^ We describe a novel, minimally invasive, ultrasound guided technique to successfully remove a dropped gallstone, causing recurrent infection, from a patient who had undergone laparoscopic cholecystectomy.

On reviewing the current literature, there are several options in the management of dropped stones. The mainstay is drainage of sepsis and complete removal of all stones. Antibiotic therapy and drainage alone, without stone removal, often results in recurrence of abscess.^8, 9^

Surgical options for stone removal include laparoscopy, providing a less invasive approach.^4^ However, the majority of reported surgical cases underwent laparotomy and surgical exploration for stone retrieval. This is often due to the deep and inaccessible location of the stones.^5^ This increases the risks of complications such as seroma, haematoma, wound dehiscence or hernia which can be as high as 20%.^10^ This is also complicated by prolonged post-operative recovery. In our case, the stone and surrounding collection was inseparable from the liver, significantly increasing the risks of liver injury/capsular tear and bleeding.

There are few previous reported cases of percutaneous stone removal, all of which have been performed under fluoroscopic guidance to identify the calculus.^11–13^ However, there are no previous cases in the literature where only ultrasound guidance has been used to locate and retrieve the gallstone, where the stone cannot be visualised with fluoroscopy.

In our case, the gallstone, although radiopaque on CT, could not be differentiated from the liver at fluoroscopy. Only 15% of gallstones are radiopaque on plain film or fluoroscopy. These stones are often the more radiodense pigment type.^14^ Performing the procedure using only ultrasound does present several technical challenges. Firstly, two operators are required to carry out the procedure: one to hold the ultrasound and sheath and the other to retrieve the stone with the basket. It also takes time for the abdominal collection to drain and the percutaneous tract to maturate. In our case, 7 days was sufficient for this. This is consistent with the other literature on the subject.^11–13^

## Learning points

1. Dropped gallstones can provide a diagnostic and therapeutic challenge for both the surgeon and radiologist. This is often due to their inconspicuous nature and late presentation of complications.
2. Removal of retained stones is essential in patient management to ensure resolution of symptoms.
3. There are several options for extraction, but surgical removal carries significant risks of morbidity and mortality as well as a prolonged post-operative recovery.
4. Here, we present a novel, minimally invasive, successful technique that could be employed to retrieve radiolucent calculi that are not visualised at fluoroscopy.
